# Editorial: Adverse health consequences of excessive smartphone usage, volume II

**DOI:** 10.3389/fpubh.2022.984398

**Published:** 2022-08-09

**Authors:** Uichin Lee, Paul H. Lee

**Affiliations:** ^1^School of Computing, KAIST, Daejeon, South Korea; ^2^Department of Health Sciences, University of Leicester, Leicester, United Kingdom

**Keywords:** excessive smartphone usage, problematic smartphone usage, adverse health consequences, anxiety and negative emotion, emotional and physical wellbeing problems

Excessive smartphone usage has diverse negative social, emotional, and physical wellbeing consequences and may lead to self-control and productivity problems as reviewed in Buscha and McCarthy (1). This special topic on *Adverse health consequences of excessive smartphone usage, volume II* covers the latest research results on anxiety, negative emotion, and physical health issues.

## Anxiety

Problematic smartphone use is related to anxiety and depression. Jiang's work in this collection investigated college students' anxiety during COVID-19 pandemics. This work identified the positive relationship between problematic social media usage and anxiety, and the mediating role of psychological capital (e.g., self-efficacy and resilience). Students' level of academic burnout moderated this relationship, with a stronger correlation among burnt-out students. Dai et al.'s work deepened our understanding of social anxiety by conducting a qualitative study. They offered detailed patterns of how college students experienced nomophobia (fear of being disconnected), phubbing (preferring online communications to face-to-face communications), and fear of missing out on social media usage. Among these anxiety-inducing factors, Servidio et al. studied how self-construal (i.e., independent vs. interdependent) is related to the fear of missing out, and its relationship with problematic phone usage. Their findings showed that interdependent self-construal was positively related to the fear of missing out and problematic phone usage.

## Emotional wellbeing

Bai et al. studied the relationship between social media usage and subjective wellbeing (e.g., emotion, satisfaction) and the mediating role of boredom proneness. They found that problematic social media usage has a negative impact on a user's subjective wellbeing, and boredom proneness mediated the relationship between problematic social media usage and subjective wellbeing. Park further studied people with visual impairment and showed that types of smartphone use are related to emotion and loneliness; for example, leisure or information search are positively related to negative emotion, but communication showed the opposite effect. Wang et al. studied the mediating role of negative emotion on the relationship between perceived stress and problematic smartphone usage among medical college students in China. Their study revealed that perceived stress and negative emotions were positively related to problematic smartphone usage.

## Physical wellbeing

This topic collection also includes two papers that reported the negative consequences of physical wellbeing (e.g., headaches, sleep disturbances, gastrointestinal problems, and dry eye symptoms). Reer et al. studied the relationship between problematic phone usage and emotional wellbeing (i.e., stress, anxiety, and loneliness) and the relationship between emotional wellbeing and physical symptoms such as headaches and sleep disturbances. Their work clearly showed that problematic smartphone usage is positively associated with both negative emotional wellbeing (i.e., higher loneliness, stress, and anxiety) and physical wellbeing (i.e., frequent headaches, and sleep disturbances). Abusamak et al. examined digital eye strain symptoms during the COVID-19 pandemics. Their findings showed that digital device usage has significantly increased during pandemics, which are positively related to physical symptoms such as headaches and neck/shoulder pain. Furthermore, usage duration is positively related to the severity of eye symptoms and the possibility of developing new eye complaints.

While problematic phone usage has various negative consequences as reported in this collection, Moshe et al. showed that mobile phone sensing offers new opportunities to proactively deal with various wellbeing issues. Recent smartphones are equipped with various sensors (e.g., GPS, sound, light) and interaction logging features (e.g., phone usage). Their work showed that applying data mining and machine learning techniques allow us to infer and predict potential negative consequences (e.g., depression and anxiety). This kind of smartphone sensing offers new opportunities for enabling just-in-time intervention for negative social, emotional, and physical wellbeing situations as illustrated in Lee et al. (2).

## Author contributions

UL wrote the draft. PL reviewed and provided feedback. All authors contributed to the article and approved the submitted version.

## Funding

This work was supported by the Basic Science Research Program through the National Research Foundation of Korea (NRF) funded by the Korean Government (MSIT) (2020R1A4A1018774 and 2022R1A2C2011536).

## Conflict of interest

The authors declare that the research was conducted in the absence of any commercial or financial relationships that could be construed as a potential conflict of interest.

## Publisher's note

All claims expressed in this article are solely those of the authors and do not necessarily represent those of their affiliated organizations, or those of the publisher, the editors and the reviewers. Any product that may be evaluated in this article, or claim that may be made by its manufacturer, is not guaranteed or endorsed by the publisher.
